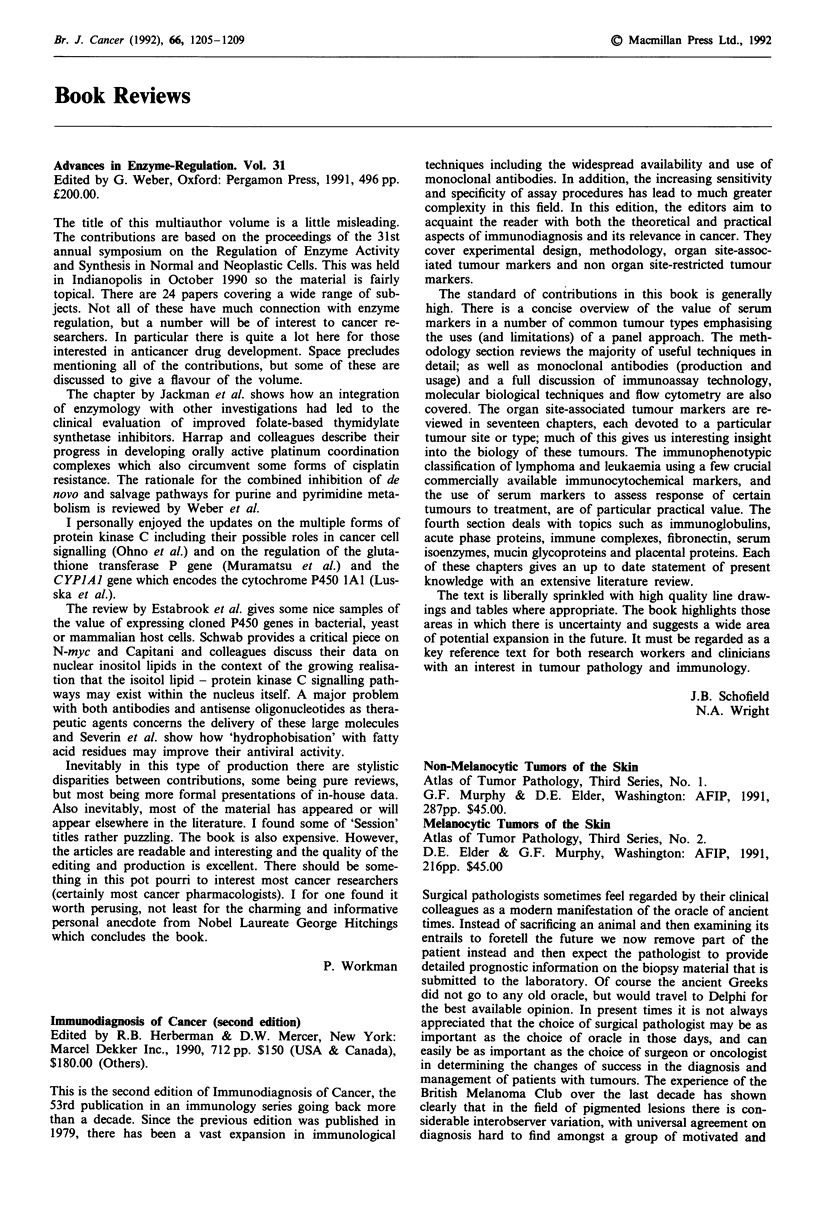# Immunodiagnosis of Cancer (2 ed.)

**Published:** 1992-12

**Authors:** J.B. Schofield, N.A. Wright


**Immunodiagnosis of Cancer (second edition)**
Edited by R.B. Herberman & D.W. Mercer, New York: Marcel Dekker Inc., 1990, 712 pp. $150 (USA & Canada), $180.00 (Others).

This is the second edition of Immunodiagnosis of Cancer, the 53rd publication in an immunology series going back more than a decade. Since the previous edition was published in 1979, there has been a vast expansion in immunological techniques including the widespread availability and use of monoclonal antibodies. In addition, the increasing sensitivity and specificity of assay procedures has lead to much greater complexity in this field. In this edition, the editors aim to acquaint the reader with both the theoretical and practical aspects of immunodiagnosis and its relevance in cancer. They cover experimental design, methodology, organ site-associated tumour markers and non organ site-restricted tumour markers.

The standard of contributions in this book is generally high. There is a concise overview of the value of serum markers in a number of common tumour types emphasising the uses (and limitations) of a panel approach. The methodology section reviews the majority of useful techniques in detail; as well as monoclonal antibodies (production and usage) and a full discussion of immunoassay technology, molecular biological techniques and flow cytometry are also covered. The organ site-associated tumour markers are reviewed in seventeen chapters, each devoted to a particular tumour site or type; much of this gives us interesting insight into the biology of these tumours. The immunophenotypic classification of lymphoma and leukaemia using a few crucial commercially available immunocytochemical markers, and the use of serum markers to assess response of certain tumours to treatment, are of particular practical value. The fourth section deals with topics such as immunoglobulins, acute phase proteins, immune complexes, fibronectin, serum isoenzymes, mucin glycoproteins and placental proteins. Each of these chapters gives an up to date statement of present knowledge with an extensive literature review.

The text is liberally sprinkled with high quality line drawings and tables where appropriate. The book highlights those areas in which there is uncertainty and suggests a wide area of potential expansion in the future. It must be regarded as a key reference text for both research workers and clinicians with an interest in tumour pathology and immunology.

J.B. Schofield
N.A. Wright